# Bladder Paraganglioma Associated With Succinate Dehydrogenase A Mutation Presenting as Pelvic Pain

**DOI:** 10.1210/jcemcr/luac026

**Published:** 2023-01-11

**Authors:** Gurbir Hehar, Dalia Rahmon, Ajaz Banka

**Affiliations:** Beaumont Hospital Royal Oak, Royal Oak, MI 48073, USA; Oakland University William Beaumont School of Medicine, Rochester, MI, USA; Beaumont Hospital Royal Oak, Royal Oak, MI 48073, USA

**Keywords:** paraganglioma, pheochromocytoma, SDHA, familial paraganglioma syndrome

## Abstract

A 21-year-old female presented to the hospital with acute onset left pelvic pain that began while urinating. Ultrasound of the pelvis revealed a 1.7 cm structure within the bladder wall. Follow-up imaging with magnetic resonance imaging confirmed a 1.9 cm mass in the urinary bladder wall. Cystoscopy with transurethral resection was performed. Histopathology of the obtained tissue confirmed the diagnosis of paraganglioma. Laboratory evaluation revealed evidence of catecholamine excess with elevated urine norepinephrine, urine normetanephrine, and plasma free normetanephrine. Functional imaging with Ga-DOTATATE positron emission tomography-computed tomography (PET-CT) revealed increased uptake in the region of the known mass without findings of metastasis. Genetic testing revealed succinate dehydrogenase A mutation, consistent with paraganglioma syndrome 5. The patient was treated with alpha-adrenergic blockade prior to partial cystectomy. Urinary bladder paraganglioma is a rare entity. The diagnosis requires a high index of clinical suspicion due to variable presentation. Hypertension and other signs of catecholamine excess, especially in relation to micturition, are important clues. Despite evidence of catecholamine excess in most patients with bladder paraganglioma, the majority are diagnosed after biopsy, indicating a need for improved diagnostic strategies in this patient population. Early diagnosis and treatment are essential to prevent potentially lethal cardiac complications and tumor metastasis.

Pheochromocytomas and paragangliomas (PPGLs) are rare tumors derived from the embryonic neural crest. They are composed of chromaffin cells of the adrenal medulla or extra-adrenal paraganglion and can secrete epinephrine, norepinephrine, and dopamine [1]. PPGLs are indistinguishable based on histologic findings and are classified according to their adrenal or extra-adrenal locations [1].

Patients with PPGLs classically present with hypertension and symptoms of catecholamine excess, such as paroxysmal headache, diaphoresis, and palpitations [2]. However, there remains a high prevalence of asymptomatic and normotensive patients, especially in familial cases.

The overall incidence of PPGLs is about 0.6 cases per 100 000 person-years [1]. The incidence has increased nearly 5-fold from 1977 to 2015, with many cases diagnosed as adrenal “incidentalomas” [2]. Diagnosis of sympathetic PPGLs involves both anatomical localization and evidence of catecholamine excess [1]. Elevated plasma fractionated metanephrines have greater sensitivity and specificity compared to fractionated catecholamines, vanillylmandelic acid, or homovanillic acid [3]. Imaging includes either computed tomography scan or T2-weighted magnetic resonance imaging (MRI) of the abdomen and pelvis and is typically performed following detection of catecholamine excess [3].

Genetic testing plays an important role in the evaluation of patients with PPGL. PPGL is associated with multiple endocrine neoplasia type 2, von Hippel-Lindau disease, neurofibromatosis type 1, and hereditary pheochromocytoma and paraganglioma [1]. Genetic carrier testing should be offered to first-degree relatives of patients diagnosed with PPGL. Similarly, biochemical screening is recommended for patients with a family history of PPGL or germline mutations associated with increased risk of developing PPGL.

## Case Presentation

A 21-year-old female presented to the emergency department with acute onset left lower quadrant abdominal pain that began while urinating. She noted the pain was worse with movement but endorsed no alleviating factors. The patient had a history of dysmenorrhea and attention deficit hyperactivity disorder treated with atomoxetine.

### Diagnostic Assessment

**Figure 1.**
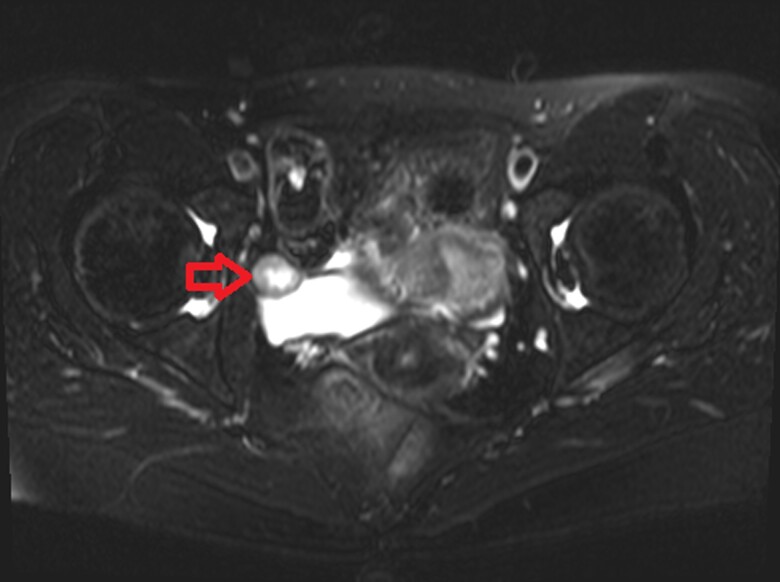
Magnetic resonance imaging pelvis with and without gadolinium. 1.9 × 1.6 × 1.3 cm well-circumscribed, heterogeneously T2 hyperintense, T1 isointense to muscle, enhancing lesion with peripheral diffusion restriction, and central cystic degeneration is seen within the right superior lateral aspect of the bladder wall.

On initial assessment, the patient was in no acute distress and vital signs were within normal limits. Ultrasound of the pelvis with doppler did not reveal signs of ovarian torsion. There was an incidental finding of a 1.7 × 1.6 × 1.5 cm echogenic structure within the bladder wall with internal vascularity. MRI of the pelvis with and without gadolinium was performed for further evaluation of the incidental bladder mass. It revealed a well-circumscribed enhancing mass measuring up to 1.9 cm located in the right superior lateral aspect of the urinary bladder (Fig. 1).

Cystoscopy with transurethral resection was performed and was complicated by significant bleeding of the mass, requiring cauterization. The patient remained hemodynamically stable. As a result of the bleeding, the resection was incomplete. Histopathology of the obtained tissue demonstrated typical morphology of paraganglioma, which was confirmed with immunohistochemistry.

**Figure 2.**
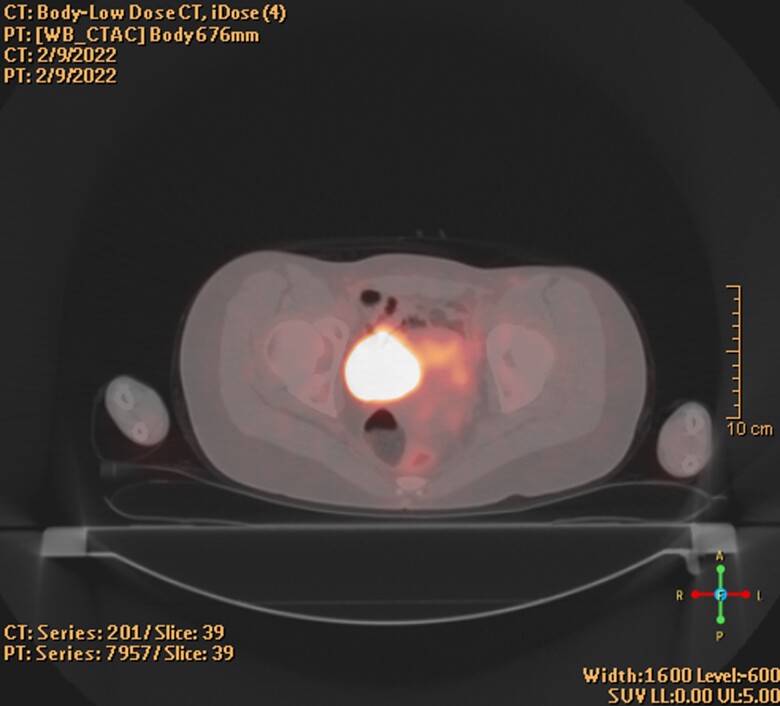
Positron emission tomography-computed tomography scan with DOTATATE staging. Increased tracer uptake in the right superolateral region of the urinary bladder.

Laboratory evaluation, performed after cystoscopy and biopsy, revealed evidence of catecholamine excess with urine norepinephrine 160 µg/24 hours (946 nmol/24 hours, ref range: 15-80 µg/24 hours), urine normetanephrine 754 µg/24 hours (4116 nmol/24 hours, ref range: 88-444 µg/24 hours), and plasma free normetanephrine 2.4 nmol/L (0.44 µg/L, ref range: < 0.90 nmol/L). Functional imaging with Ga-DOTATATE PET-CT revealed increased uptake in the region of the known mass without findings of metastasis (Fig. 2). Genetic testing revealed c.91C > T (p.R31*) mutation in 1 copy of the succinate dehydrogenase A (SDHA) gene, consistent with paraganglioma syndrome 5. Immunohistochemical staining of obtained tissue showed loss of SDHB. Staining for SDHA and genetic testing were not performed.

### Treatment

The patient was surgically treated with robotically assisted laparoscopic partial cystectomy of the bladder mass. Prior to surgery, alpha-adrenergic blockade was initiated with doxazosin 0.5 mg nightly and beta blockade with metoprolol tartrate 12.5 mg twice daily. To adjust therapy, the patient was instructed to keep a daily blood pressure log. Doxazosin was increased to a dose of 1 mg nightly to achieve a goal blood pressure of less than 130/80 mmHg.

### Outcome and Follow-Up

Surgical resection was completed without complication. The patient is scheduled to follow with endocrinology for ongoing tumor surveillance. Genetic testing revealed SDHA mutation in 2 out of 3 first-degree relatives.

## Discussion

Herein, we present a rare case of a young woman who initially presented with pelvic pain and was found to have a functional paraganglioma of the bladder with pathogenic mutation in SDHA, consistent with familial pheochromocytoma/paraganglioma syndrome.

Paraganglioma of the bladder is extremely rare, accounting for less than 0.05% of all bladder tumors and about 0.7% of all paragangliomas [4]. This patient was diagnosed at the age of 21, relatively early compared to the mean age at presentation of 50 years [4]. The clinical presentation of functional bladder paraganglioma is variable, but the most common presenting symptoms include hypertension, headache, hematuria, palpitations, and syncope. Symptoms associated with micturition, so-called micturition attacks, are reported in up to 53% of patients [4]. Most patients with bladder paraganglioma present with either symptoms of catecholamine excess (30%) or urinary/pelvic symptoms (29%) including pelvic pain [4]. Incidental finding of functional bladder paraganglioma is less common, occurring in 3% to 10% of cases [4]. This patient presented with pelvic pain that began during urination and did not seem to have hypertension or evidence of catecholamine excess. She initially presented with a blood pressure of 129/74 mmHg. Repeat measurement during an office visit 4 days later was 116/70 mmHg. Importantly, although the patient was not found to have hypertension, ambulatory blood pressure monitoring was not performed. Therefore, paroxysmal hypertension (including hypertension associated with micturition) was not ruled out.

Historically, pheochromocytoma and paraganglioma were thought to be hereditary in only 10% of cases. With novel genetic testing and the identification of more than 20 susceptibility genes, more recent studies suggest that the incidence of hereditary disease is about 40% [5]. Patients with hereditary disease are known to present a decade earlier on average compared to sporadic cases. Guidelines recommend consideration of genetic testing for all patients with PPGL and testing for succinate dehydrogenase (SDH) mutations specifically in patients with paraganglioma [6]. Mutations in the SDH subunit genes (SDHA, SDHB, SDHC, SDHD) and a cofactor gene (SDHAF2) are the most common cause of hereditary PPGL and are responsible for most cases [5].

This patient had apparently sporadic disease with only a single tumor and no known family history. However, the patient displayed atypical features of sporadic disease including young age and highly unusual tumor location. Genetic evaluation revealed pathogenic mutation in SDHA, associated with paraganglioma syndrome 5. The mutation in this case [(c.81C > T (p. R31*)] is a missense mutation and is the most frequent type of SDHA mutation. A recent study of 61 patients with bladder PGL who underwent genetic testing found that only 2 patients had mutations in SDHA [4]. This case is unique because, unlike most patients with SDHA mutations who present with head and neck paraganglioma [7], this patient had sympathetic paraganglioma of the bladder. Interestingly, mutations in SDHA are also found in healthy control populations, suggesting that the penetrance of this mutation is relatively low. Although mutation in SDHB was not identified, resected paraganglioma tissue revealed loss of SDHB staining, strongly suggesting positive germline SDHB mutation in this patient.

Imaging to search for additional paragangliomas or metastatic disease should be performed in all patients with known metastatic disease and in certain patients with risk for metastasis including extra-adrenal tumor, large tumor size, or disease-causing genetic mutation [6]. Functional imaging, including I-MIBG scintigraphy, F-FDG PET-CT, F-DOPA PET-CT, and Ga-DOTATATE PET-CT, is effective for this purpose. To evaluate for metastatic or recurrent disease, patients found to have SDHA or SDHB mutation should undergo either MRI of the skull base and neck, thorax, retroperitoneum, and pelvis or Ga-DOTATATE PET-CT. After tumor removal, MRI of the surgical region should be performed annually for the first 3 years [1]. For body regions without tumor, MRI should be performed every 3 years. Biochemical testing should be performed yearly.

Cystoscopy is frequently the initial investigation in patients with a bladder mass. Bladder paraganglioma are highly vascular and can bleed extensively. Surgical manipulation can result in “catecholamine storm,” which may manifest as hemodynamic instability, hypertensive crisis, cardiac arrhythmia, coronary spasm, pulmonary edema, or stroke [8]. The abrupt decrease in serum catecholamines following tumor resection can precipitate sudden and profound hypotension, which is poorly responsive to adrenergic agonists [8]. Therefore, accurate presurgical diagnosis is critical to decisions regarding alpha blockade prior to intervention and type of surgery. This proves challenging for bladder paraganglioma as these tumors are rare and have variable clinical presentations. As a result, most patients are diagnosed after biopsy [4]. Patients should be evaluated for clinical clues such as paroxysmal hypertension and other symptoms associated with catecholamine excess, especially in association with micturition.

If paraganglioma is considered in the differential diagnosis, biochemical testing should be performed prior to surgery. Most patients with bladder paraganglioma have evidence of catecholamine excess; however, up to 30% of patients in a recent study did not [4]. Once functional paraganglioma has been diagnosed biochemically, preoperative blockade to prevent adrenergic crisis is recommended. Guidelines recommend alpha-adrenergic receptor blockers for 7 to 14 days prior to surgery as well as a high sodium diet and fluid intake to reverse volume contraction and prevent hypotension after tumor removal [6]. Alpha blockade is recommended even for normotensive patients to prevent unpredictable hypertension during surgery.

Surgical resection is the standard of care for PPGL. Because these tumors are highly vascular, secrete catecholamines, and generally located deep in the bladder mucosa, partial cystectomy is often preferred. Other treatment options include transurethral resection (TUR) and radical cystectomy. In this case, pelvic pain was the only apparent presenting symptom of bladder paraganglioma. Definitive diagnosis did not occur until after cystoscopy with biopsy. In patients without a presurgical diagnosis of paraganglioma, the majority undergo TUR [9]. In contrast, most patients with a presurgical diagnosis undergo cystectomy with only 5% of patients undergoing TUR. Furthermore, patients with a presurgical diagnosis are less likely to experience hypertensive crisis or preoperative cardiovascular incident. This highlights the importance of presurgical diagnosis with regards to surgical planning and prevention of intraoperative cardiovascular events.

## Learning Points

- Paraganglioma is a rare but important type of tumor to consider in the differential diagnosis of a bladder mass.
- Hypertension and other signs of catecholamine excess, especially in relation to micturition, are important clues to diagnosis of bladder paraganglioma.
- Despite evidence of catecholamine excess in the majority of patients with bladder paraganglioma, most are diagnosed after biopsy, indicating a need for improved awareness and diagnostic strategies in this patient population.
- Early diagnosis and treatment of paraganglioma are essential to prevent potentially lethal cardiac complications and tumor metastasis.

## Data Availability

Data sharing is not applicable to this article as no datasets were generated or analyzed during the current study.
